# Generation and characterization of a Cre-inducible ZNF768 overexpression mouse model

**DOI:** 10.1038/s41598-025-03110-8

**Published:** 2025-06-05

**Authors:** Audrey Poirier, Laura Tribouillard, Manal Kordahi, Yves Gélinas, Joanny Roy, Marie-Josée Beaulieu, Michèle Orain, Marie-Renée Blanchet, Philippe Joubert, Mathieu Laplante

**Affiliations:** 1 Centre de Recherche de l’Institut Universitaire de Cardiologie et de Pneumologie de Québec (CRIUCPQ), Université Laval, Québec, QC Canada; 2 Centre de Recherche sur le Cancer de l’Université Laval, Université Laval, Québec, QC Canada; 3 Faculté de Médecine, Université Laval, Québec, QC Canada

**Keywords:** Cancer models, Physiology

## Abstract

Zinc-finger protein 768 (ZNF768) is an emerging transcription factor regulating cell proliferation and senescence. Although the role of ZNF768 in regulating cell fate decision has been demonstrated in vitro, its importance in controlling physiological and pathophysiological processes in vivo is still unclear. Here, we report the generation of a transgenic mouse model allowing the conditional overexpression of ZNF768. This was achieved by inserting an inverted *Znf768* coding sequence surrounded by heterologous Cre recognition sites in the *Gt(ROSA)26Sor* mouse locus (*FLExZnf768*). To study the impact linked to systemic overexpression of ZNF768, mice carrying the *FLExZnf768* allele were crossed with CMV-Cre mice to produce a whole-body ZNF768 transgenic mouse (WB-ZNF768-Tg). As expected, WB-ZNF768-Tg mice showed higher ZNF768 levels in various tissues. These mice were born at the expected Mendelian ratio and did not display apparent phenotypes. Because ZNF768 levels are often overexpressed in cancer, we assessed tumor development in WB-ZNF768-Tg mice. However, ZNF768 overexpression was not sufficient to promote 3-methylcholantrene-induced fibrosarcoma and KRAS^G12D^-induced lung adenocarcinoma in mice. Overall, we report the generation of a conditional mouse for ZNF768 overexpression and reveal that forcing ZNF768 expression is not sufficient to alter tumour development in mice.

## Introduction

Cells are constantly exposed to various stresses that challenge their integrity and function including genotoxic, replicative, oxidative, and proteotoxic stress^1,2^. In response to these insults, damaged cells activate a range of adaptive responses to promote repair, resolve stress, and ensure survival^3,4^. Alternatively, when damages cannot be resolved, senescence or apoptosis can be triggered to prevent propagation of injured cells^5,6^. Complex signaling networks that monitor the nature and extent of damages have evolved to enable cells to decide between these different fates^7^. Failure to properly regulate this decision can lead to aberrant cell proliferation and tumor development^1^.

Recent studies have identified Zinc finger protein 768 (ZNF768) as a new factor involved in the regulation of cell fate decision^8,9^. ZNF768 is a protein highly conserved in mammals. This transcription factor contains C_2_H_2_ domains and localizes in the nucleus^8,9^. Interestingly, ZNF768 also contains amino acid stretches in N-terminal that resemble the heptad repeats found in the C-terminal domain (CTD) of the large subunit of RNA polymerase II (RPB1). It was recently reported that genotoxic and oncogenic stresses promote ZNF768 phosphorylation in these stretches, an effect that triggers its degradation by the proteasome^9^. Supporting the importance of ZNF768 in promoting cell proliferation, it was reported that ZNF768 depletion causes apoptosis and senescence, while its overexpression is sufficient to bypass oncogene-induced senescence^9^. Mechanistically, ZNF768 binds to genomic regions called Mammalian-wide interspersed repeats (MIRs) to regulate the expression of numerous genes, including a “core” set of genes controlling the cell cycle^8,9^. Additionally, we showed in cancer cells that ZNF768 interacts with tumor suppressor p53 to repress its phosphorylation and activation^9,10^. Together, these observations identify ZNF768 as an emerging transcription factor that integrates stress signals to control cell proliferation^11^.

Following its identification, ZNF768 was found to be overexpressed in a wide variety of human cancers^9,12^. Analysis of public databases revealed that *ZNF768* amplification and increased expression are frequent in multiple cancer types^9^. Tissue microarray analyses using a cohort of patients with lung cancer confirmed that elevated ZNF768 protein expression is common in lung adenocarcinomas^12^. In this cancer, ZNF768 levels positively correlate with high proliferative features such as Ki-67 and the mitotic score^12^. Together, these results suggest that ZNF768 overexpression could be a key event participating to tumorigenesis.

To explore the physiological role of ZNF768 in vivo, a complete knockout mouse model has been generated and characterized^13^. We showed in previous work that ZNF768 null mice are viable and show only a mild growth defect early in life^13^. In response to irradiation, knockout mice show increased signs of radiosensitivity and altered gene expression in tissues. This change in the acute transcriptional response to irradiation affects a subset of p53 target genes but also has a broader impact on genes regulating transmembrane receptor signaling, cell adhesion, and growth. The role of ZNF768 in tumorigenesis was also investigated in this model. In these studies, we found that ZNF768 loss was sufficient to repress lung tumor development in mice with hyperactive RAS^13^. Altogether, these results support previous observations in cells and reveal a functional impact of ZNF768 loss on tumour development in vivo.

As the physiological roles of ZNF768 in health and disease are only emerging, there is a need to develop additional tools to study this protein in vivo. Here, we report the generation of a transgenic mouse model allowing the conditional overexpression of ZNF768 in cell types and tissues. This was achieved by inserting an inverted *Znf768* coding sequence surrounded by heterologous Cre recognition sites in the *Gt(ROSA)26Sor* (also known as *Rosa26*) mouse locus (*FLExZnf768*). Highlighting the versatility of this model, we show conditional overexpression of ZNF768 in adult tissues and primary cells following exposure to the Cre recombinase. Here, we show that whole-body ZNF768 overexpression is tolerated and minimally affects development in mice. We also show that forcing ZNF768 expression is not sufficient to promote tumor development in carcinogen- and oncogene-driven cancer mouse models. With this work, we provide the scientific community a versatile model to study ZNF768 in vivo, and we reveal that ZNF768 is not a primary driver of cancer in mice.

## Results

### Development and production of a mouse carrying a *FLExZnf768* allele

**Fig. 1:**
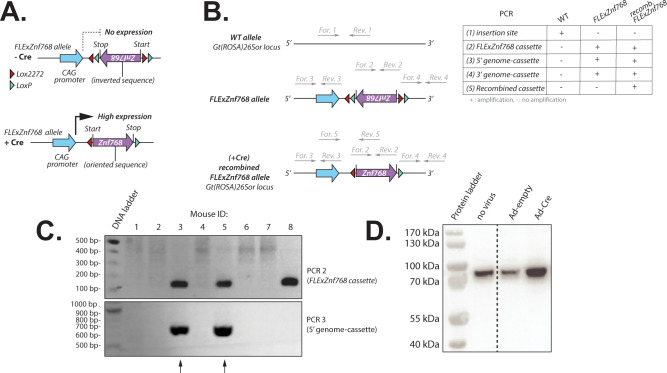
Development and production of a mouse carrying a *FLExZnf768* allele. (**A**) Schematic overview of the *FLExZnf768* transgenic allele. The *FLExZnf768* transgenic allele contains the *Znf768* coding sequence in the inverted orientation flanked by two pair of heterologous Cre recognition sites (LoxP and Lox2272) in the opposite direction with an alternate order at both ends of the transgene. In the presence of Cre, the LoxP and Lox2272 sites undergo recombination with their respective sites, resulting in the inversion of the *Znf768* coding sequence, which allows *Znf768* transgene expression under the control of the CAG promoter. (**B**) Genotyping strategy to confirm the insertion and orientation of the *FLExZnf768* cassette into the *Gt(ROSA)26Sor* locus by PCR. PCR 1 amplifies a 199 bp fragment at the insertion site in the WT mice. When the cassette is inserted, the primers used for PCR1 become too far apart to generate an amplicon with the PCR conditions used. PCR 2 amplifies a 168 bp fragment within the *FLExZnf768* cassette. PCR 3 and 4 amplify a 700 bp and a 938 bp fragment flanking the 5’ and 3’ insertion site-cassette junction respectively. PCR 5 amplifies a 461 bp fragment of the *FLExZnf768* cassette only after Cre-mediated recombination. Only primer pairs that produce an amplicon within 70 s at a rate of 1 kb per minute are presented on the DNA sequences. (**C**) PCR genotyping of founder mice to confirm the insertion and the orientation of the *FLExZnf768* cassette. PCR 2 and 3 are shown. Arrows point to individuals that harbor the cassette in the right orientation. (**D**) Western blot showing the expression of ZNF768 in TTFs isolated from *FLExZnf768* mice that were transduced or not with Ad-empty or Ad-Cre virus for 24 h.

To investigate the impact of ZNF768 overexpression in vivo, we have generated a conditional ZNF768 overexpressing mouse model using the flip-excision (FLEx) system, which uses directional recombination vectors to control transgene expression^14^. These mice carry a *FLExZnf768* allele which was incorporated in the safe harbor locus *Gt(ROSA)26Sor* using the CRISPR-Cas9 technology. This cassette is composed of the mouse *Znf768* coding sequence in the inverted orientation flanked by two pair of heterologous Cre recognition sites (LoxP and Lox2272) in the opposite direction with an alternate order at both ends of the transgene (Fig. 1A). The allele is preceded by a cytomegalovirus (CMV) early enhancer/chicken β actin (CAG) promoter and followed by a polyA sequence (Fig. 1A). In the presence of Cre, the recombinase catalyzes reversion of the gene cassette through recombination between either of the double recognition sites. It then catalyzes deletion of the intervening sequence between the same two recognition sites and produces the reversed cassette with different recognition sites at both ends preventing further recombination. We next designed a strategy to confirm the insertion and the orientation of the gene cassette in the *Gt(ROSA)26Sor* locus (Fig. 1B). Upon generation of the founder mice, offsprings were screened for the insertion of the *FLExZnf768* cassette (Fig. 1C). This design resulted in the successful generation of the hemizygous *FLExZnf768* line of mice in a C57BL/6N background. To confirm the validity of the construct and the transgene expression under the presence of the Cre recombinase, we have isolated tail tip fibroblasts (TTFs) from *FLExZnf768* hemizygous mice and treated the cells with Cre-expressing adenovirus (Ad-Cre). As expected, treatment of *FLExZnf768* TTFs with Ad-Cre resulted in an increase in ZNF768 protein levels, confirming the validity of the construct (Fig. 1D).

### Production of whole-body ZNF768 overexpresser mice

**Fig. 2:**
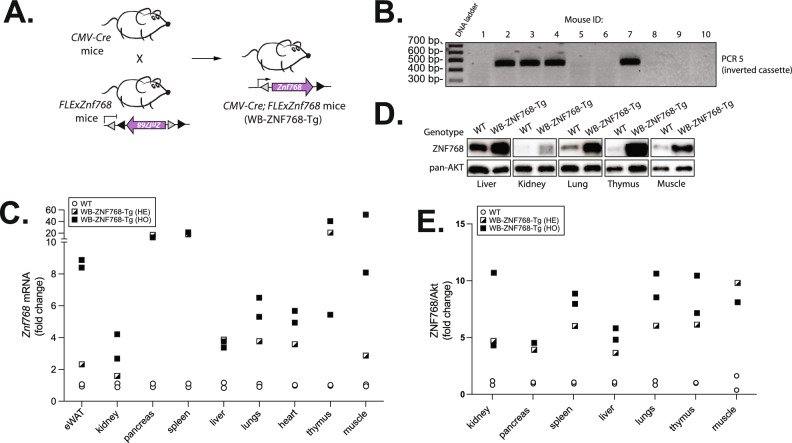
Production of WB-ZNF768-Tg mice. (**A**) Breeding strategy to produce the WB-ZNF768-Tg mice. *FlexZnf768* mice were crossed with CMV-Cre mice to induce the recombination in all tissues, including germ cells. (**B**) Genotyping of founder mice confirming the recombination of the *FLExZnf768* cassette. PCR 5 is presented (see Fig. 1B for details). (**C**) qPCR analysis of *Znf768* transcript levels in the tissues of WB-ZNF768-Tg hemizygous and homozygous mice (*n* = 1–2 animals/genotype). (**D**) Western blot showing the expression of ZNF768 in the tissues of WB-ZNF768-Tg homozygous mice. Pan-Akt was used as a loading control. (**E**) Quantification of ZNF768 protein levels in in the tissues of WB-ZNF768-Tg hemizygous and homozygous mice (*n* = 1–2 animals/genotype).

Following the generation of the *FLExZnf768* conditional mouse model, we produced a whole-body ZNF768 overexpresser mice to validate our construct and study the impact of ZNF768 overexpression in vivo. Briefly, *FLExZnf768* mice were crossed with a CMV-Cre mouse strain which expresses the Cre recombinase under the control of a CMV promoter to generate *CMV-Cre; FLExZnf768* mice, hereinafter referred to as whole-body ZNF768 transgenic mice (WB-ZNF768-Tg) (Fig. 2A). The CMV-Cre mice express the Cre before implantation during early embryogenesis leading to recombination in all tissues and cell types, thus driving the systemic expression of the *Znf768* transgene in mice. As previously shown, because the CMV promoter drives Cre expression in germ cells, the recombined allele is heritable and passed to the next generation^15,16^. Mice carrying one recombined *FLExZnf768* allele were bred in the C57BL/6J background to remove the recombinase transgene and only keep the recombined *FLExZnf768* cassette.

We first used the strategy described in Fig. 1B to confirm the recombination of the *FLExZnf768* cassette in different tissues. As observed in Fig. 2B, the PCR reactions confirmed the recombination of the cassette in the WB-ZNF768-Tg mice. We next measured the levels of *Znf768* mRNA (Fig. 2C) and ZNF768 protein (Fig. 2D) expression in different tissues to assess the level of overexpression. As expected, higher ZNF768 levels were observed in various tissues of hemizygous and homozygous WB-ZNF768-Tg mice relative to non-transgenic mice (Fig. 2C–E). While higher expression of ZNF768 protein was observed in some tissues of WB-ZNF768-Tg mice carrying 2 alleles, we found that one allele of the transgene was sufficient to achieve overexpression ranging from 3- to 10-fold in the tissues tested (Fig. 2E). For this reason, hemizygous mice were then used for the rest of this study.

### WB-ZNF768-Tg mice are viable and do not display apparent phenotypes

**Fig. 3:**
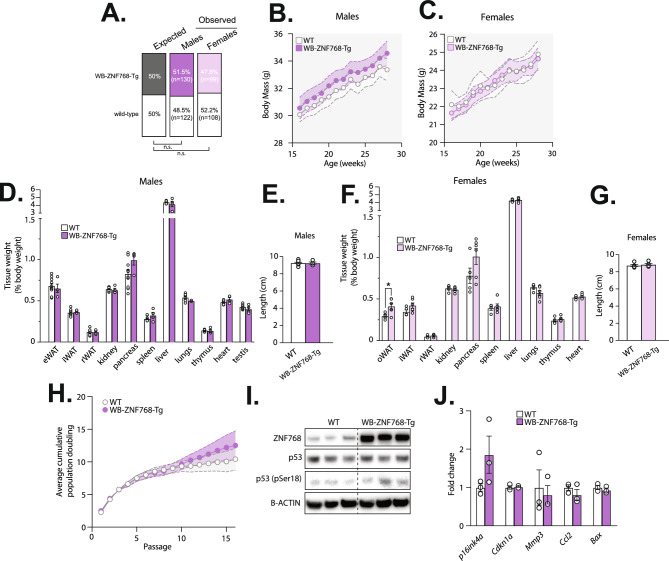
WB-ZNF768-Tg mice are viable and do not display apparent phenotypes. (**A**) Mendelian ratios calculated from the crossing of WB-ZNF768-Tg heterozygotes with wild-type mice (*n* = 459). Significance was determined by binominal test. (**B**-**C**) Body weight (*n* = 8–11 mice/group), (**D** and **F**) tissue weight (% of body weight) (*n* = 4–10 mice/group), and (**E** and **G**) length (*n* = 4–10 mice/group) of WB-ZNF768-Tg and wild-type male and female mice. Mice were sacrificed at 10–12 weeks of age. Data represent the mean ± SEM. For body weight, significance was determined by 2-way ANOVA. For tissue weight and length, significance was determined by 2-tailed, unpaired t test. *P < 0.05 versus controls. (**H**) Growth curve of MEFs collected from wild-type or WB-ZNF768-Tg mice cultured under the standard 3T3 protocol. This experiment was reproduced three times in independent MEFs lines and pooled results are presented (*n* = 10 MEFs/group). Data represent the mean ± SEM. Significance was determined by 2-way ANOVA. (**I**) Western Blot showing the expression of ZNF768, total and phosphorylated p53 in WB-ZNF768-Tg homozygous and wild-type MEFs. B-ACTIN was used as a loading control. Representative samples are shown. (**J**) RT-qPCR analysis of gene expression in MEFs (*n* = 3 cell lines/group). Data represent the mean ± SEM. Significance was determined by 2-tailed, unpaired t test.

WB-ZNF768-Tg hemizygous mice were born at the expected Mendelian ratios, were viable, and appeared phenotypically normal (Fig. 3A). Initial characterization of the WB-ZNF768-Tg mice showed that ZNF768 overexpression did not induce any apparent phenotype in mice. As presented in Fig. 3B,C, these mice did not show significant difference in body weight from 16 to 28 weeks of age. In addition, no difference in body length was observed between the WB-ZNF768-Tg and wild-type mice in both sexes (Fig. 3E,G). At sacrifice, we found that most tissues from male and female WB-ZNF768-Tg weighed the same compared to wild-type mice (Fig. 3D,F). Altogether, these results show that ZNF768 overexpression has minimal effects on development and physiology in mice.

The results presented above suggest that ZNF768 is not sufficient to promote cell proliferation and changes in organ size when overexpressed in mice. To directly test the effect of ZNF768 overexpression on cell proliferation in our model, we have collected MEFs from control and WB-ZNF768-Tg mice and we have measured proliferation capacity using a standard 3T3 approach. As presented in Fig. 3H, we found no change in proliferation in response to ZNF768 overexpression. Also, we observed no difference in total and phosphorylated p53 levels (Fig. 3I), and no effect on the expression of several p53 target genes (Fig. 3J). These observations indicate that in primary cells, ZNF768 overexpression does not affect proliferative capacity and the activity of p53. These results align with previous findings showing that transduction of ZNF768 in human primary fibroblasts is not sufficient to accelerate proliferation in vitro^9^.

### Tumor susceptibility in ZNF768 overexpressing mice

Aberrant expression of ZNF768 has been observed in multiple types of cancer in mice and humans^9,12^. Furthermore, several lines of evidence suggest that ZNF768 may promote cancer cell proliferation and tumor development^9,12,13^. To explore the oncogenic potential of ZNF768, we assessed spontaneous tumor susceptibility in control and WB-ZNF768-Tg during a period of 20 months. Over this time, we observed no spontaneous tumors in WB-ZNF768-Tg mice, indicating that sustained ZNF768 overexpression is not sufficient to initiate and promote tumor development in mice.

**Fig. 4:**
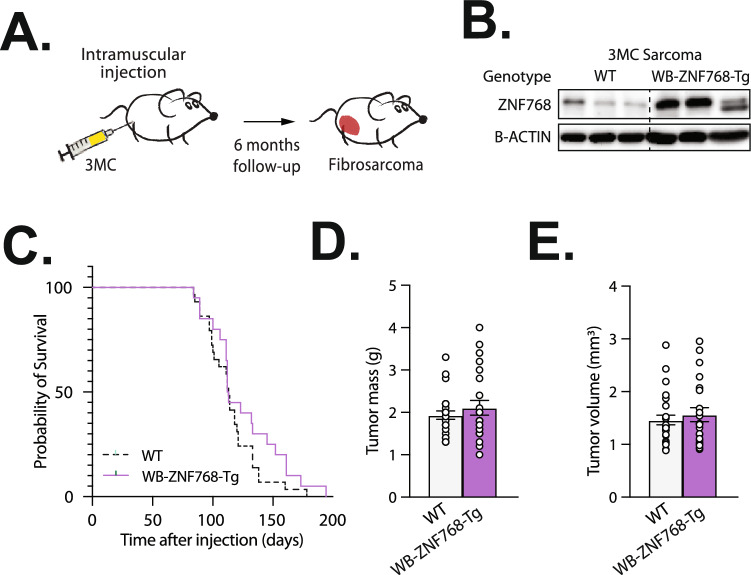
Overexpression of ZNF768 does not alter 3MC-induced sarcoma in mice. (**A**) Schematic representation of the strategy used to induce sarcoma in mice. 3-methylcholanthrene (3MC) is injected in the hindlimb of WB-ZNF768-Tg and wild-type mice. Using this approach, mice develop high grade sarcoma with short latency (tumors in red). (**B**) Western blot of ZNF768 protein levels in 3MC sarcoma of wild-type and WB-ZNF768-Tg mice. B-ACTIN is used as a control. (**C**) Kaplan–Meier plot of wild-type and WB-ZNF768-Tg mice of both sexes injected with 3MC (*n* = 20–29 mice/group). Significance was determined by Log-rank test. (**D**) Mass and (**E**) volume of wild-type and WB-ZNF768-Tg 3MC sarcoma at sacrifice (*n* = 20/29 mice/group). Data represent mean ± SEM. Significance was determined by 2-tailed, unpaired t test.

We then assessed whether ZNF768 overexpression could promote tumor development in carcinogen- and oncogene-induced cancer mouse models. First, wild-type and ZNF768 overexpressing mice were injected in the hind limb with 3-methylcholanthrene (3MC), a carcinogen that induces fibrosarcoma development (Fig. 4A). Mice were then followed for approximately 6 months and tumor development was observed. First, overexpression of ZNF768 in fibrosarcoma of WB-ZNF768-Tg mice was confirmed (Fig. 4B). Here, no difference in the survival was observed between control and transgenic mice (Fig. 4C). Supporting these findings, the mass and volume of the tumors at sacrifice did not differ between the genotypes (Fig. 4D,E).

**Fig. 5:**
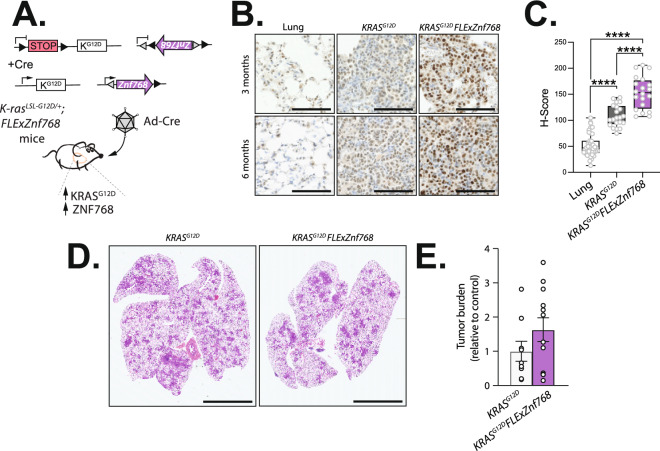
Overexpression of ZNF768 does not alter lung tumor development driven by oncogenic KRAS^G12D^. (**A**) Schematic representation of the strategy used to overexpress ZNF768 in lung tumors induced by oncogenic KRAS^G12D^. The *KRAS*^*G12D*^ and *KRAS*^*G12D*^; *FLExZnf768* mice carry the conditional activatable *LSL-KRAS*^*G12D*^ allele. The silenced LSL allele comprises the *KRAS* gene carrying a point mutation (G12D) whose expression is blocked by the presence of a LoxP-flanked stop codon. The *KRAS*^*G12D*^; *FLExZnf768* mice also contain the *FLExZnf768* allele which contains the *Znf768* mRNA sequence in the antisense orientation flanked by two pair of heterologous Cre recognition sites (LoxP and Lox2272) in the opposite direction with an alternate order at both ends of the transgene. Following the intratracheal injection of Ad-Cre virus, the Cre-mediated recombination excises the stop codon of the *LSL-KRAS*^*G12D*^ cassette and permits the oncogenic protein to be expressed leading to the development of lung lesions ranging from atypical adenomatous hyperplasia to adenocarcinoma. The Cre also allows the recombination of the *FLExZnf768* cassette, which allows *Znf768* transgene overexpression. (**B**) Immunohistochemistry of normal lung and tumors isolated from *KRAS*^*G12D*^ and *KRAS*^*G12D*^;*FLExZnf768* mice 3 and 6 months after Ad-Cre administration. (magnification × 20, 100 µm scale bars). (**C**) Quantification of ZNF768 immunochemistry staining using the H-score in lung and lung tumors of *KRAS*^*G12D*^ and *KRAS*^*G12D*^; *FLExZnf768* mice 6 months after Ad-Cre delivery (*n* = 20–40 regions/group). Data are presented as box plots, where the center line represents the median, the box extends from the first to the third quartile and the whiskers indicate the minimum and maximum values. Significance was determined by 1-way ANOVA. ****P < 0.0001 versus controls. (**D**) Representative H&E staining of lung sections from *KRAS*^*G12D*^ and *KRAS*^*G12D*^; *FLExZnf768* mice 6 months after Ad-Cre administration (magnification × 0.5, 5 mm scale bars). (**E**) Quantification of tumor burden in lung sections of *KRAS*^*G12D*^ and *KRAS*^*G12D*^; *FLExZnf768* mice described in (**D**) (*n* = 9–12 mice/group). Significance was determined by 2-tailed, unpaired t test.

In a second set of experiments, we took advantage of a well-described model of lung cancer to test the role of ZNF768 overexpression on carcinogenesis. Briefly, mice carrying an unrecombined *FLExZnf768* allele were crossed with mice carrying a Lox-Stop-Lox (LSL) termination sequence with the KRAS^G12D^ mutation (*LSL-KRAS*^*G12D*^). As previously demonstrated, intratracheal administration of Ad-Cre in *LSL-KRAS*^*G12D*^ mice promotes the recombination of the LSL cassette, which allows the expression of oncogenic KRAS^G12D^ and the development of lung tumors in mice^17^. As illustrated in Fig. 5A, intratracheal administration of Ad-Cre in mice carrying the *FLExZnf768* and the *LSL-KRAS*^*G12D*^ alleles allows the concomitant overexpression of ZNF768 and oncogenic KRAS^G12D^ in the lungs. These mice are referred as *KRAS*^*G12D*^*;FLExZnf768* mice. To confirm ZNF768 overexpression in response to Ad-Cre delivery in lungs, ZNF768 levels were measured in the tumors 3 and 6 months post-transduction using immunohistochemical staining of the lung and quantified using the H-score method, as described previously^12^. As shown before^9^, we observed an increase in ZNF768 levels in lung tumors compared to healthy lung tissue (Fig. 5B,C). Also, *KRAS*^*G12D*^;*FLExZnf768* mice showed higher levels of ZNF768 in the tumors at both time points, confirming the efficiency of our approach (Fig. 5B,C). However, despite elevated ZNF768 expression, no difference in tumor burden was observed between the 2 genotypes at 6 months following Ad-Cre administration (Fig. 5D,E).

## Discussion

Studies published by independent groups showed that ZNF768 depletion severely impairs proliferation and induces senescence in multiple cell lines in vitro^8,9,12^. This transcription factor was reported to bind MIR regions to regulate a vast array of genes, including several cell cycle effectors^8,9,12^. Additionally, ZNF768 was shown to bind p53, an effect that blocks p53 phosphorylation and reduces its activity^9,10^. The rapid degradation of ZNF768 in response to replicative and oncogenic stress suggests that, in normal cells, ZNF768 acts as control point to regulate proliferative capacity^11^. Following this model, we proposed that the elevation in ZNF768 levels observed in most human cancers may represent a strategy developed by neoplastic cells to evade senescence and support proliferation^9,11^. We recently reported the generation of a ZNF768 knockout mouse model to study the role of ZNF768 in tumorigenesis^13^. Using this tool, we showed that ZNF768 loss significantly reduced lung tumor burden driven by oncogenic KRAS^G12D^ in mice^13^. Although these observations confirm that ZNF768 exerts pro-oncogenic functions in vivo, it remains unclear whether enhanced ZNF768 expression is sufficient to drive tumorigenesis.

Here, we report the generation of a transgenic mouse model allowing the conditional overexpression of ZNF768 in mice. Using various approaches, we confirmed higher ZNF768 expression in tissues and primary cells collected from transgenic mice. Herein, we tested whether ZNF768 overexpression could drive tumor development in basal conditions or in carcinogen- and oncogene-induced cancer mouse models. Systemic overexpression of ZNF768 in mice did not induce spontaneous tumors in mice. Moreover, no difference in tumorigenesis was observed between wild-type and ZNF768 overexpressing mice in response to 3MC or oncogenic KRAS^G12D^. Although these results suggest that ZNF768 per se is insufficient to drive tumour development in mice, it is possible that adaptive mechanisms might have been triggered to counteract the effect of ZNF768 overexpression. It was previously reported that ZNF768 is phosphorylated on multiple residues with many of these sites falling within, or near to the heptad repeats stretches in the N-terminal end of ZNF768^9^. As demonstrated in RPB1, these repeats possess the capacity to encode information via combinatorial phosphorylation. In RPB1, extensive phosphorylation and post-translational modification of these repeats impact the recruitment of chromatin regulators and RNA processing factors at specific stages of transcription^18^. Interestingly, several phosphomotifs found in the heptad repeats of ZNF768 are phosphorylated in response to oncogenic RAS^G12V^, with some promoting its degradation by the proteasome^9^. Whether phosphorylation of these residues and other post translational modifications on ZNF768 could have altered its activity and counteracted the effect of its systemic overexpression is a possibility that remains to be explored. It is also worth noting that all the in vivo experiments presented here were performed with transgenic mice carrying only one recombined *FLExZnf768* allele. Although ZNF768 was expressed at high levels in these mice, the use of homozygous *FLExZnf768* mice showing even higher ZNF768 expression might have been required to impact tumor development. Beyond these technical considerations, it is also important to take into consideration that mutations and genetic events occurring in pre-neoplastic cells can remain latent for a long time and become effective drivers only at a particular stage of tumorigenesis or in conjunction with specific mutations. Here, two cancer mouse models were used where we looked at tumor size as an indicator of tumor development at specific time points. Expanding these studies to additional cancer mouse models, different strain backgrounds with higher oncogenic potential, and broader tumor characteristics may reveal novel functions or impacts of ZNF768 overexpression on tumorigenesis.

Overexpression is a powerful tool to study gene function and can reveal novel roles not found in traditional loss of function studies. The development of a conditional ZNF768 overexpressing mouse model brings a plethora of possibilities to study the functions of ZNF768 in vivo along with the previously described ZNF768 knockout mouse model^13^. In comparison to ZNF768 null mice, this new model might be better suited to study the role of ZNF768 in physiological processes where ZNF768 is degraded in vivo, such as in response to DNA damaging agents. The FLEx strategy used to develop our mouse model has also been successfully used to control multiple genes simultaneously, a system termed FLEx switch. These genetic switches can conditionally turn off the expression of one gene, while simultaneously turning on the expression of another. Among their many uses, FLEx switches can serve to conditionally rescue gene knockout, introduce conditional point mutation or functional mutations in vivo or can be paired with florescent reporter proteins to track genetically modified cells^14,19–22^. Combining the use of our novel *FLExZnf768* allele with other transgenes offer endless options to study the functions of ZNF768 in different experimental contexts.

In conclusion, we report the generation of a transgenic mouse model allowing the conditional overexpression of ZNF768. We show that whole-body ZNF768 overexpression does not impact mouse physiology and is not sufficient to promote tumor development in carcinogen- and oncogene-driven cancer mouse models. With this work, we provide the scientific community with a versatile model to study the impact of ZNF768 expression in both health and disease, which is a key step towards a better understanding of the roles and functions of this intriguing protein.

## Material and methods

### Mouse models

The *FLExZnf768* cassette is composed of the mouse *Znf768* coding sequence (NCBI Reference Sequence NM_146202.1, nucleotide 79 to 1785) in the antisense orientation with the Kozak sequence located upstream of the start codon. This sequence is flanked by two pairs of heterologous Cre recognition sites (LoxP and Lox2272) in the opposite direction with an alternate order at both ends of the transgene. The allele is preceded by a cytomegalovirus (CMV) early enhancer/chicken β actin (CAG) promoter and followed by a polyA sequence. Mice carrying the *FLExZnf768* cassette in the *Gt(ROSA)26Sor* locus were generated by the McGill Integrated Core for Animal Modeling (MICAM) using the CRISPR-Cas9 technology. All experiments followed the guidelines of the Canadian Council of Animal Care and were approved by McGill University’s Animal Care Committee. A guide RNA (gRNA) previously reported to be efficient for editing the mouse *Gt(ROSA)26Sor* locus was chosen to insert the *FLExZnf768* cassette^23–25^. The sequence of this gRNA, localized on chr6:113,076,040–113,076,05 GRCm38/mm10, is ACTGGAGTTGCAGATCACGA (with PAM motif GGG). Guide RNA was synthesized using a T7 transcription kit (Thermofisher, #AM1354). As a template for homologous directed recombination, we constructed the *FLExZnf768* cassette flanked by standard *Gt(ROSA)26Sor* homology regions extending 300 bp upstream and downstream of the CRISPR-Cas9 cut site. The gRNA and mRNA Cas9 and templates were subsequently utilized for microinjection into mouse zygotes derived from C57BL/6N mice at a concentration of 20 ng/µl each. These embryos were then implanted into pseudopregnant CD-1 females for gestation, resulting in the generation of founder mice. CMV-Cre and LSL-*KRAS*^*G12D*^ mice were obtained from The Jackson Laboratory (strain #006054 and #008179 respectively) and have been described previously^16,17^. WB-ZNF768-Tg mice were produced by crossing the CMV-Cre (JAX, strain #006054) and *FLExZnf768* mice. Mice carrying one recombined *FLExZNF768* allele were bred in the C57BL/6J background to remove the Cre-recombinase transgene. LSL-*KRAS*^*G12D*^*; FLExZnf768* mice were generated by crossing LSL-*KRAS*^*G12D*^ (JAX, strain #008179) and *FLExZnf768* mice. In all experiments, mice were euthanized by cervical dislocation under isoflurane anesthesia. Experiments with mice were performed in accordance with relevant guidelines and regulations, including the with ARRIVE guidelines (https://arriveguidelines.org).

### Genotyping

Genotyping of *FLExZnf768* and WB-ZNF768-Tg mice was performed with the following primers (forward, reverse): (1) insertion site in the *Gt(ROSA)26Sor* locus (GAAGACTCCCGCCCATCTTC, GTAAGGGAGCTGCAGTGGAG), (2) *FLExZnf768* cassette (TCACCATCGACCCTCTAGTC, GGTATTTGTGAGCCAGGGCAT), (3) 5′ genome-cassette junction (GCTACAGCCTCGATTTGTGG, ATCATGAAGCCCCTTGAGCA), (4) 3′ genome-cassette junction (CTCACCTCGACCCATGGTAA, TCGTCGTCTGATTGGCTCTC), and (5) recombined *FLExZnf768* cassette (TCTGTGGGAAGTCTTGTCCC, TCCGACCTCATCCGCCA). Using an elongation step of 1 min at a rate of 1 kb per minute, PCR 1 amplifies a 199 bp fragment at the insertion site in the WT mice only. PCR 2 amplifies a 168 bp fragment within the *FLExZnf768* cassette in both orientations. PCR 3 and 4 amplify a 700 bp and a 938 bp fragment flanking the 5′ and 3′ insertion site-cassette junction respectively. PCR 5 amplifies a 461 bp fragment of the *FLExZnf768* cassette only present after Cre-mediated recombination.

### Isolation and culture of primary TTFs

Tail tip fibroblasts (TTFs) were isolated from 1–2 mm piece of tail collected from a *FLExZnf768* mouse generated by our group, as described above in the Material and Methods section. Hair was removed with scissors and the tissue was minced with a sterile razor blade/scalpel in collagenase type 1 (4 mg/ml in DMEM) (Worthington, # LS004196). Minced tissue was incubated for 30 min at 37 *°*C and then seeded into a TC100 culture dish. The cell lines were cultured in complete Dulbecco’s Modified Eagle Medium (DMEM) (Wisent, #319-005-CL) supplemented with Fetal Bovine Serum (FBS) (10%) (Sigma-Aldrich, #F1051) and penicillin–Streptomycin (1%) (Wisent, #450-201-EL). Cells were transduced at multiplicity of infection (MOI) 100 with Ad5-Cre virus (Viral Core Facility, University of Iowa, USA, #5) for 24 h in the presence of 8 µg/ml polybrene.

### Isolation and culture of primary MEFs

Mouse embryonic fibroblasts (MEFs) were derived from E13.5 embryos generated by the crossing of hemizygous WB-ZNF768-Tg mice. Cells were isolated and cultured as described before^13^. The 3T3 protocol was performed as described by Todaro et al.^26^ In these experiments, 20 independent MEFs lines were used (10 control and 10 lines carrying two *FLExZnf768* transgenic alleles) in 3 independent experiments.

### Animal experiments

All experimental protocols were approved by the Animal Ethics Committee of Université Laval (CPAUL) and followed the guidelines of the Canadian Council on Animal Care and the ARRIVE guidelines (https://arriveguidelines.org). 3-methylcholanthrene (3-MC) (Sigma-Aldrich, #213942) was prepared and injected as described previously^13^. Animals were monitored twice per week after onset of tumor growth was observed. Subcutaneous tumor volume was quantified by measuring the length and width of tumors using a digital caliper. The ellipsoid volume formulas (L × W^2^ × π/6) was used to estimate tumor size. Tumor size was not allowed to exceed 2 cm^3^ in volume in accordance with institutional guidelines. Lung tumor initiation with pAd5CMVCre-mCherry (Viral Core Facility, University of Iowa, USA, #649) was done as previously described^13^. Animals were monitored once per week after infection until sacrifice.

### Gene and protein expression analysis

Western blotting and quantitative real-time PCR were performed as described previously^27^. For western blotting, the following primary antibodies were used: ZNF768 (Aviva Systems Biology, FLJ23436, dilution 1:1000), p53 (1C12) [Cell Signaling Technology, #2524, dilution 1:1000], phospho p53 (Ser 15) [Cell Signaling Technology, #9284, dilution 1:1000], Akt (pan) (C67E7) [Cell Signaling Technology, #4691, dilution 1:1000] and β-actin [Cell Signaling Technology, #4967, dilution 1:1000]. Secondary antibody was purchased from Cell Signaling Technology [Cell Signaling Technology, #7074, dilution 1:5000]. For quantitative real-time PCR, the following primers were used (forward, reverse): *Znf768* (GATGTGCACAGTCCCAACG, GGGGTTCAAGCCCAAAAGGT), *p16ink4a* (TGTGCATGACGTGCGGG, TAGTGGGGTCCTCGCAGTT), *Cdkn1a* (GACAAGAGGCCCAGTACTTCC, CTTGCAGAAGACCAATCTGCG), *Mmp3* (CTCGTGGTACCCACCAAGTC, CTCGTGGTACCCACCAAGTC), *Ccl2* (CCCAATGAGTAGGCTGGAGA, TCTGGACCCATTCCTTCTTG), *Bax* (AGGATGCGTCCACCAAGAAG, CTTGGATCCAGACAAGCAGC), and *Gapdh* (GGCAAATTCAACGGCACAGT, CTCGTGGTTCACACCCATCA).

### Tissue staining and immunohistochemistry

For immunohistochemistry, samples were fixed for 24 h in 10% buffered formalin phosphate (Thermofisher, #SF100) and then processed for paraffin embedding. Five-micrometre-thick sections were cut from formalin-fixed paraffin-embedded tissue (FFPE) sections on a microtome and placed on charged slides. Staining with ZNF768 antibody was performed as described previously^12^.

### Slide digitalization and tumor analyses

Analysis of the tumors was done on digitalized slides. Whole images of each stained tissue slide were obtained at 20X magnification using a slide scanner (NanoZoomer 2.0-HT; Hamamatsu, Bridgewater, NJ, USA) and visualized using the companion software (NDP view, Hamamatsu, Japan). Tumor burden was calculated manually as the tumor area over total lung area and was assessed by a pathologist on 2 separate lung sections per mouse. Scoring of protein expression was done using an open source software for bioimage analysis (QuPath; Queen’s University, Belfast, Northern Ireland). The H-score method was used to evaluate ZNF768 expression. This score (0–300) combines the percentage of stained nuclei and the staining intensity using the following equation: (% nuclei of intensity 1 × 1) + (% nuclei of intensity 2 × 2) + (% nuclei of intensity 3 × 3).

## Statistics and reproducibility

All the statistical analyses were performed using Prism (version 10.2.3). In all panels, data represent the mean ± SEM. The statistical tests used to determine significance, and the sample size are identified in the figure legends. Key findings in cells were reproduced in at least 2 independent experiments or in different cell lines. Animal studies were performed once using several animals per groups to insure significance. Uncropped images of gels and western blots used in Fig. 1C,D, 2B,D, 3I, and 4B are presented in Supplementary Fig. 1.

## Supplementary Information

Supplementary Information.

## Data Availability

The datasets used and/or analysed during the current study available from the corresponding author on reasonable request.
