# Supplementary material for: Generation and characterization of a Cre-inducible ZNF768 overexpression mouse model
Source: Sci Rep. 2025 Jun 5;15:19792. doi: 10.1038/s41598-025-03110-8 (PMC12141517; doi:10.1038/s41598-025-03110-8)

Figure 1C

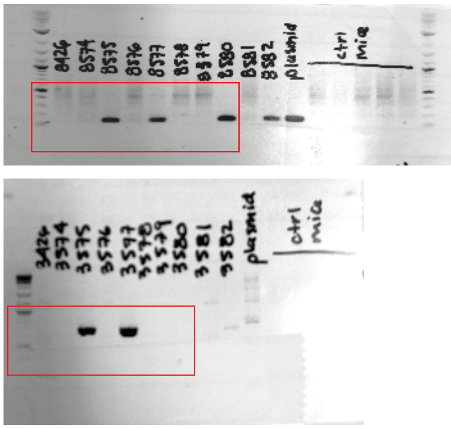

Figure 1D

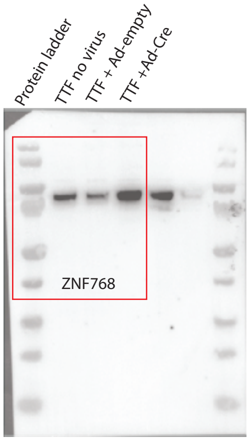

Figure 2B

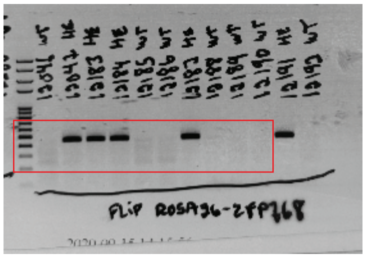

Figure 2D

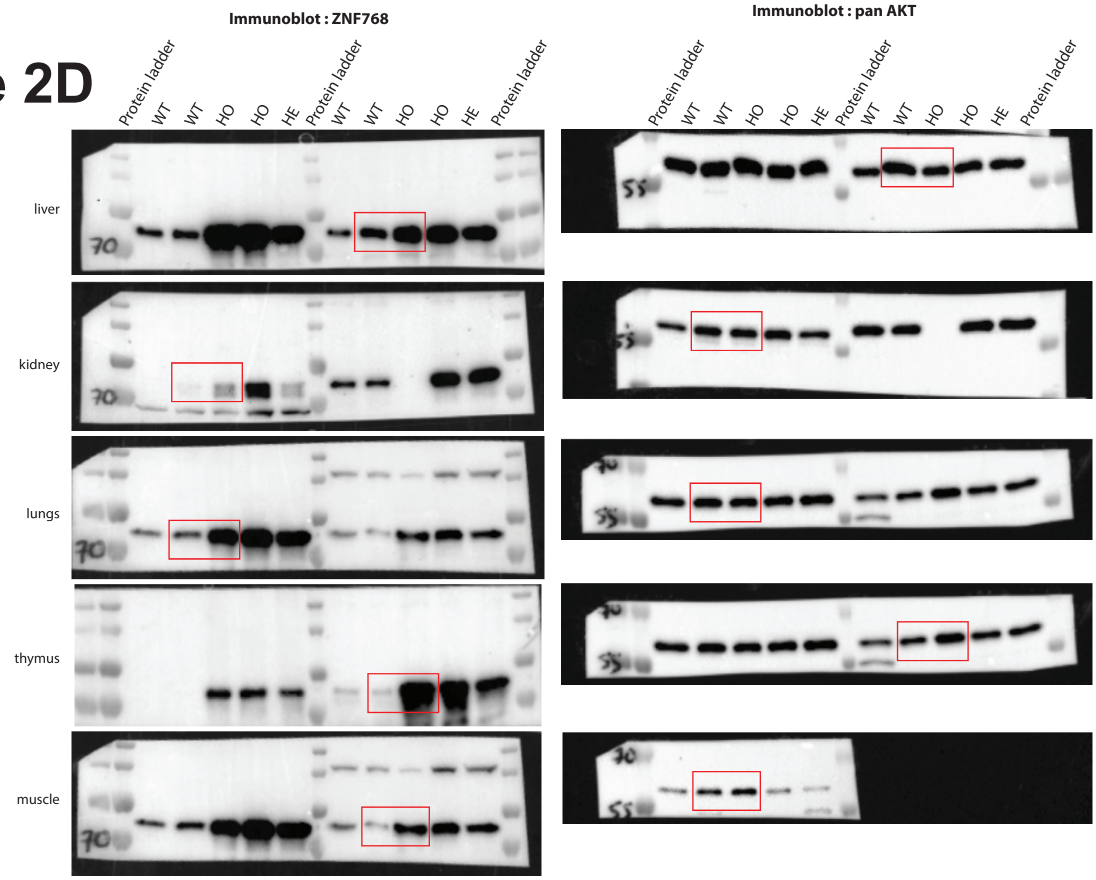

Figure 3I

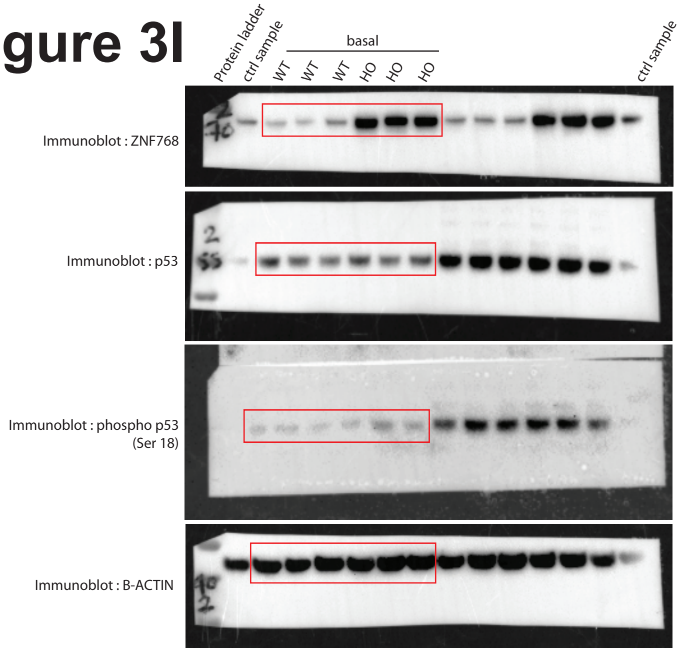

Figure 4B

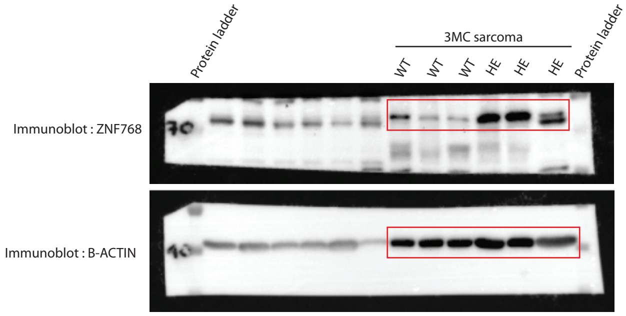

Supplement: Supplementary file 1 — Supplementary Information. [file 41598_2025_3110_MOESM1_ESM.pdf]
